# PB.25. Implementation of two new digital mammography technologies for Breast Test Wales: more or less accurate?

**DOI:** 10.1186/bcr3737

**Published:** 2014-11-03

**Authors:** T Evans, B Burlton, K Gower-Thomas

**Affiliations:** 1 Royal Glamorgan Hospital, South Wales, UK

## Introduction

The aim of this study is to compare the performance of Sectra and Hologic digital mammography, two different technologies introduced to Breast Test Wales in 2012 for breast cancer screening. Specifically, any variation in findings and whether different types of cancer - for example, invasive or non-invasive - are better identified by either system.

## Methods

This is a retrospective study of 50,000 consecutive mammograms starting from 2012 of females aged 50 to 70 carried out in the Breast Test Wales mobile screening units. All cancers identified in the two arms of the study were detailed and compared specifically with regards to type (ductal or lobular), size, grade, invasion and node status. Performance of each mammography technology with regard to the detection rate of each cancer type was analysed for any statistically significant difference.

## Results

Of 50,000 women screened, 500 cancers were detected with no significant difference in overall invasive cancer detection, invasive ductal or invasive lobular detection found between the two screening technologies. However, Hologic (1.44 per 1,000) was found to have a significantly higher non-invasive cancer detection rate than Sectra (2.88 per 1,000), *P *< 0.001.

## Conclusion

While Hologic and Sectra have been found to be comparable in terms of invasive cancer detection, this study shows a difference in the number of non-invasive cancers they detect. Further study and a decision as to whether the difference is clinically significant long term are now required.

